# Cascade Ligand- and Structure-Based Virtual Screening to Identify New Trypanocidal Compounds Inhibiting Putrescine Uptake

**DOI:** 10.3389/fcimb.2018.00173

**Published:** 2018-05-25

**Authors:** Lucas N. Alberca, María L. Sbaraglini, Juan F. Morales, Roque Dietrich, María D. Ruiz, Agustina M. Pino Martínez, Cristian G. Miranda, Laura Fraccaroli, Catalina D. Alba Soto, Carolina Carrillo, Pablo H. Palestro, Alan Talevi

**Affiliations:** ^1^Laboratory of Bioactive Compounds Research and Development (LIDeB), Medicinal Chemistry, Department of Biological Science, Exact Sciences College, National University of La Plata Buenos Aires, Argentina; ^2^Institute of Sciences and Technology Dr César Milstein (ICT Milstein), Argentinean National Council of Scientific and Technical Research (CONICET) Buenos Aires, Argentina; ^3^Department of Microbiology, Parasitology and Immunology, School of Medicine, Institute of Microbiology and Medical Parasitology (CONICET), University of Buenos Aires Buenos Aires, Argentina

**Keywords:** Chagas disease, *Trypanosoma cruzi*, putrescine uptake, drug repositioning, drug repurposing, cinnarizine, virtual screening, positive predictive value

## Abstract

Chagas disease is a neglected tropical disease endemic to Latin America, though migratory movements have recently spread it to other regions. Here, we have applied a cascade virtual screening campaign combining ligand- and structure-based methods. In order to find novel inhibitors of putrescine uptake in *Trypanosoma cruzi*, an ensemble of linear ligand-based classifiers obtained by has been applied as initial screening filter, followed by docking into a homology model of the putrescine permease *Tc*PAT12. 1,000 individual linear classifiers were inferred from a balanced dataset. Subsequently, different schemes were tested to combine the individual classifiers: MIN operator, average ranking, average score, average voting, with MIN operator leading to the best performance. The homology model was based on the arginine/agmatine antiporter (AdiC) from *Escherichia coli* as template. It showed 64% coverage of the entire query sequence and it was selected based on the normalized Discrete Optimized Protein Energy parameter and the GA341 score. The modeled structure had 96% in the allowed area of Ramachandran's plot, and none of the residues located in non-allowed regions were involved in the active site of the transporter. Positivity Predictive Value surfaces were applied to optimize the score thresholds to be used in the ligand-based virtual screening step: for that purpose Positivity Predictive Value was charted as a function of putative yields of active in the range 0.001–0.010 and the Se/Sp ratio. With a focus on drug repositioning opportunities, DrugBank and Sweetlead databases were subjected to screening. Among 8 hits, cinnarizine, a drug frequently prescribed for motion sickness and balance disorder, was tested against *T. cruzi* epimastigotes and amastigotes, confirming its trypanocidal effects and its inhibitory effects on putrescine uptake. Furthermore, clofazimine, an antibiotic with already proven trypanocidal effects, also displayed inhibitory effects on putrescine uptake. Two other hits, meclizine and butoconazole, also displayed trypanocidal effects (in the case of meclizine, against both epimastigotes and amastigotes), without inhibiting putrescine uptake.

## Introduction

World Health Organization (WHO) describes neglected tropical diseases (NTDs) as a group of tropical diseases which mainly affect people living in poverty, lacking adequate sanitary conditions and in close contact with the infectious vectors (World Health Organization, 2015). One the most important NTDs—in numerical terms—is Chagas disease, a parasitic disease endemic to Latin-America, caused by the infection by the protozoan *Trypanosoma cruzi*. This parasite can be transmitted to humans and more than 150 domestic and wild mammals, making complete eradication of the parasite practically impossible. The main route of transmission of *T. cruzi* is through the feces of the insect vector, known as *vinchuca*, a bug of the subfamily Triatominae. There are also other transmission routes, as congenital transmission and blood transfusions (Rassi et al., 2010), which are becoming increasingly important in the last years. WHO estimates based on 2010 data indicate that more than 6 million people are infected with *T. cruzi* worldwide, mostly in Latin-American countries (World Health Organization, 2015). However, several reports suggest that the actual number of infected people could be quite higher, reaching 10 million people (Ventura-Garcia et al., 2013; Stanaway and Roth, 2015; Browne et al., 2017).

Chagas disease presents two clinical phases. The initial or acute phase, which lasts between 4 and 8 weeks, is in general asymptomatic or might present as a self-limiting febrile illness. After the acute phase, an indeterminate, latent phase follows, with absence of clinical symptoms. About 60–70% of these people will remain in the indeterminate phase, but the remaining 30–40% will develop the symptomatic chronic phase characterized by damage to specific organs—particularly heart, esophagus, or colon. The chronic phase remains throughout life drastically reducing life expectancy among these patients (Nunes et al., 2013).

The only two approved drugs for the treatment of Chagas disease so far are Benznidazole and Nifurtimox, launched in the early 1970s. Both compounds are well-tolerated in children and effective during the acute phase. However, they present considerable side effects in adults, different susceptibility among *T. cruzi* strains and limited efficacy in adults in chronic phase (Morillo et al., 2015; Bermudez et al., 2016).

Drug repositioning (also known as drug repurposing, indication expansion and indication shift) represents an interesting strategy to approach the development of new medications for NTD (Ekins et al., 2011; Bellera et al., 2015; Ferreira and Andricopulo, 2016; Sbaraglini et al., 2016). It consists in finding novel medical uses for existing drugs, including approved, experimental, discontinued and shelved drugs. Drug repurposing has several advantages over the search of *de novo* drugs. Since the new indication is built on already available pharmacokinetic and safety data, drug development time and costs can be considerably shortened. Possible manufacturing issues have also been solved. There are several successful cases of repositioned drugs in the field of NTDs: the anticancer drug eflornithine has been approved for the treatment of sleeping sickness and the antifungal drug amphotericin B has been repurposed for treatment of visceral leishmaniasis. To date, however, although there are several reports of drug candidates to be repositioned for the treatment of Chagas disease, none of these has yet been approved (Andrews et al., 2014; Klug et al., 2016; Sbaraglini et al., 2016). While initially drug repurposing stories arose from serendipitous observations, the drug discovery community has progressively adopted more systematic approximations to indication expansion (Ekins et al., 2011; Jin and Wong, 2014; Ferreira and Andricopulo, 2016), including genomic and structural biology tools, *in silico* screening and high-throughput screening platforms.

**Figure 1:**
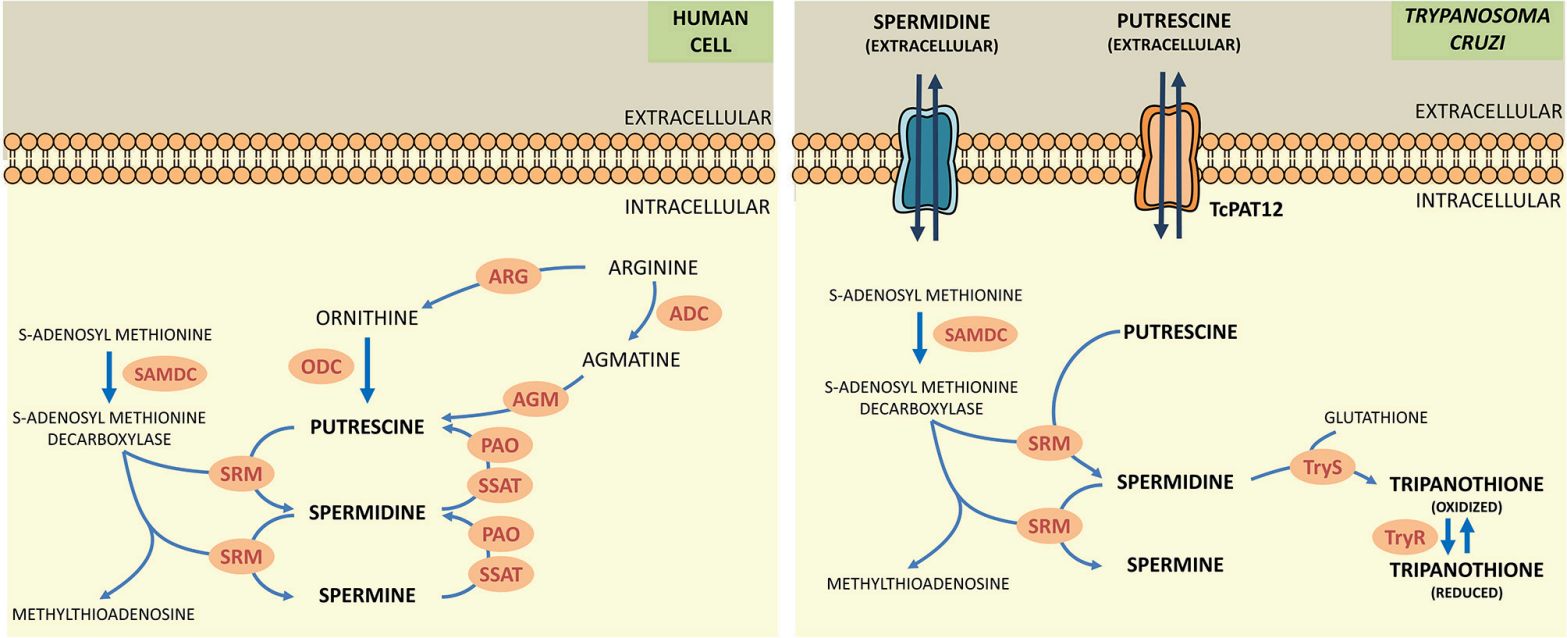
Comparative scheme of polyamine metabolism in human cells and *T. cruzi*. ARG, arginase; ADC, arginine decarboxylase; AGM, agmatinase; ODC, ornithine decarboxylase; SAMDC, s-adenosylmethionine decarboxylase; SRM, spermidine synthase; PAO, polyamine oxidase; SSAT, spermidine acetyltransferase; TryS, trypanothione synthetase; TryR, trypanothione reductase.

Polyamines (putrescine, spermidine, spermine) are low molecular weight polycations with crucial physiologic role in all the eukaryotic cells. They take part in fundamental cellular processes such as growth, differentiation, macromolecular biosynthesis and protection against oxidative damage. The polyamine metabolism in *T. cruzi* differs significantly from its human counterpart since the parasite lacks the enzymes arginine decarboxylase and ornithine decarboxylase, which are necessary for the biosynthesis of polyamines (Figure 1; Carrillo et al., 1999, 2003). Thus, *T. cruzi* depends on the incorporation of polyamines from the host cell. These functions are carried out by polyamine transporters such as the high-affinity putrescine permease *Tc*PAT12 (or *Tc*POT1.1) which does not present homologous in the mammalian lineage (Carrillo et al., 2006). The importance of polyamines for parasites survival, the inability of the parasite to biosynthesize polyamines and the exclusivity of the putrescine transporter in *T. cruzi* makes putrescine uptake an attractive target for the search of new trypanocidal drugs (Hasne et al., 2016).

Back in 2016, we reported the first *in silico* drug repurposing campaign to discover novel inhibitors of polyamine uptake in *T. cruzi* (Alberca et al., 2016); such study applied an ensemble of ligand-based models to screen DrugBank 4.0 and Sweetlead databases and resulted in the identification of three candidates that impaired putrescine transport: paroxetine, triclabendazole and sertaconazole. Here, we have improved our ligand-based computational models and complemented them with molecular docking based on a homology model of *Tc*PAT12; the combined screening has been applied to identify two new *Tc*PAT12 potential inhibitors: clofazimine and cinnarizine. We also report, for the first time, the use of Positivity Predictive Value (PPV) surfaces analysis to select the score threshold that will be used in the virtual screening procedure. The two hits were assayed against *T. cruzi* epimastigotes and trypomastigotes, and the inhibitory effect on putrescine uptake was also determined.

## Materials and methods

### Ligand-based virtual screening

#### Dataset collection

**Figure 2:**
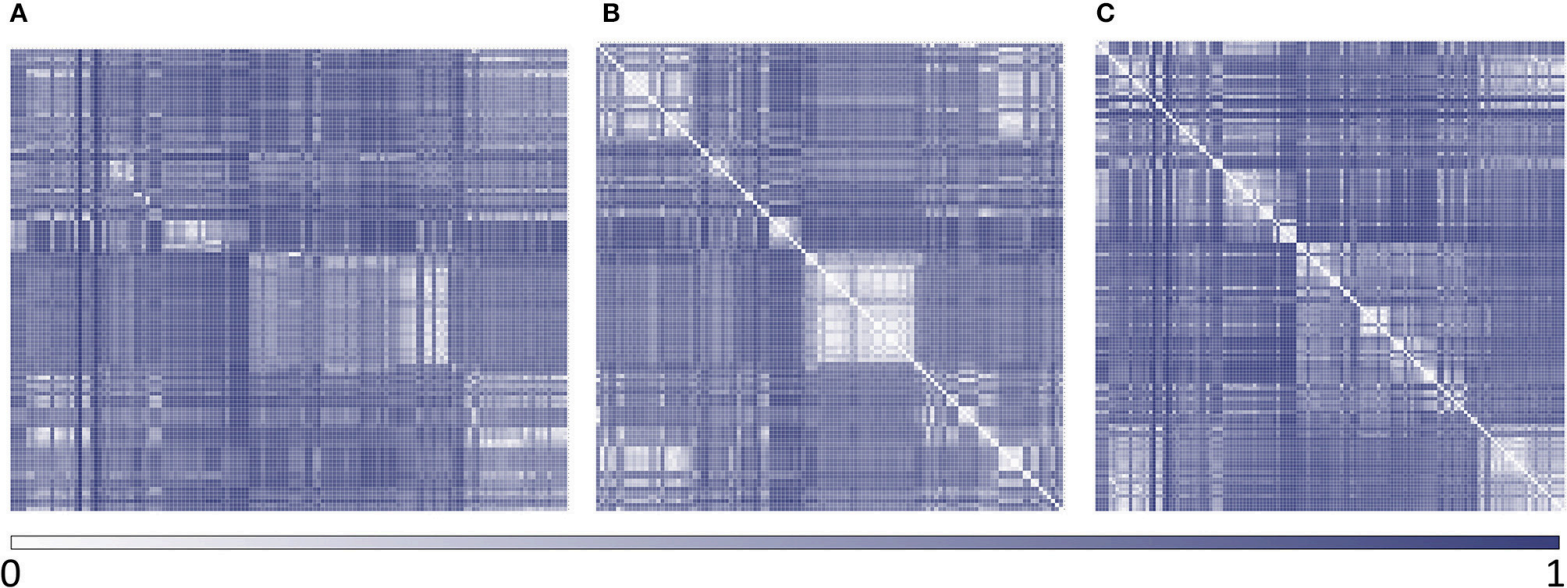
Heatmaps of dissimilarity of the whole dataset and within each category. **(A)** active compounds vs. inactive compounds; **(B)** active compounds vs. active compounds; **(C)** inactive compounds vs. inactive compounds.

Polyamine analogs previously assayed against *T. cruzi* were compiled from literature. 256 polyamine analogs were found and conformed the dataset used here for model calibration and validation. Such dataset was curated using the standardization tool available in Instant JCHEM v. 17.2.6.0. We have labeled the 256 compounds as ACTIVE or INACTIVE according to their half-maximal effective concentrations (EC_50_) against *T. cruzi*. The ACTIVE category included compounds with EC_50_ below 20 μM; the remaining compounds were included in the INACTIVE category. Considering such cut-off, the dataset includes 116 actives and 140 inactives. The molecular diversity of the whole dataset and within each category can be appreciated in the heatmaps displayed in Figure 2, which show, for every compound pair in the database, the Tanimoto distance computed using ECFP_4 molecular fingerprints. The heatmap was built using Gitools v. 2.3.1 (Perez-Lamas and Lopez-Bigas, 2011) and Tanimoto distances were calculated using ScreenMD—Molecular Descriptor Screening v. 5.5.0.1 (ChemAxon, 2002-2011). The dataset is included as Supplementary Information.

#### Dataset splitting

The resulting dataset of 256 polyamine analogs was divided into two groups using a representative sampling procedure: (a) training set, that was used to calibrate the models and; (b) test set, that was used to externally validate the models. The representative partition of the dataset resulted from a serial combination of clustering procedures: first, we have used the hierarchical clustering method included in LibraryMCS software (version 17.2.13.0–ChemAxon), which relies on the Maximum Common Substructure (MCS). From the resulting clusters, a compound from each cluster was randomly chosen and used as a seed to perform a non-hierarchical clustering using the k-means algorithm, as implemented in Statistica 10 Cluster Analysis module (Statsoft, 2011). Such procedure was performed in an independent manner for the ACTIVE and INACTIVE categories.

Seventy-five percent of the compounds in each cluster of the ACTIVE category were kept for the training set (totaling 87 compounds); an equal number of compounds were taken from the INACTIVE category clusters (62.14% of each INACTIVE cluster). The remaining 29 active and 53 inactive compounds were assigned to test set. Note that, to obtain the training set, we have under-sampled the INACTIVE category, so that a balanced calibration sample was obtained. In that way, model bias toward predicting a specific category was avoided.

#### Molecular descriptor calculation and modeling procedure

Three thousand six hundred sixty-eight conformation-independent descriptors were computed with Dragon 6.0 software. A random subspace-based method was applied to obtain 1,000 random descriptor subsets of no more than 200 potential independent variables each. In the random subspace approach, the features (independent variables, e.g., molecular descriptors) are randomly sampled and each model (learner) is trained on one subset of the feature space (El Habib Daho and Amine Chikh, 2015; Vyskovsky et al., 2016), which causes individual models not to over-focus on features that display high explanatory power in the training set.

A dummy, binary variable (class label) was used as dependent variable. Such variable was assigned observed values of 1 for compounds within the ACTIVE, and observed values of 0 for compounds in the INACTIVE category.

Using a Forward Stepwise approach and a semi-correlation approach (Toropova and Toropov, 2017), 1,000 linear classifiers were obtained, one from each of the random subsets of features. In order to avoid overfitting, only one molecular descriptor every 10 training instances was allowed into each model, with no more than 12 independent variables per model. No regression coefficient with *p*-value above 0.05 was allowed into the model.

R language and environment was used for all data analysis. The R package data table (https://cran.r-project.org/package=data.table) was used to handle datasets.

The robustness and predictive ability of the models were estimated through randomization and leave-group-out cross-validation tests. In the case of randomization, the class label was randomized across the compounds in the training set. The training set with the randomized dependent variable was then used to train new models from the descriptor selection step. Such procedure was repeated 10 times within each descriptor subset. It is expected that the randomized models with perform poorly compared to the real ones. Regarding the Leave-Group-Out cross-validation, 18-compound subsets were randomly removed from the training set in each cross-validation round and the model was regenerated. The resulting model was used to predict the class label for the 18 removed compounds. The procedure was repeated 10 times, removing each of the training set compounds once.

#### Ensemble learning

Classifier ensembles are known to handle complex data and to provide better generalization and accuracy than single model classifiers (El Habib Daho and Amine Chikh, 2015; Carbonneau et al., 2016; Min, 2016; Vyskovsky et al., 2016); they can be particularly useful to prevent overfitting when handling datasets that suffer from small sample size while their dimensionality is large (Vyskovsky et al., 2016), a quite frequent scenario in the drug design field.

The best individual classifiers were selected and combined (selective ensemble learning approach) using the area under the ROC curve metric (AUC ROC) as criterion of performance. Systematic combinations of the 2–100 best performing classifiers were analyzed. Four combination schemes were applied to obtain a combined score: MIN operator (which returns the minimum score among the individual scores of the combined models); Average Score; Average Ranking and Average Voting. Voting was computed according to the equation previously used by Zhang and Muegge (2006). AUC ROCs were obtained with the pROC package (Robin et al., 2011); the Delong method was used to obtain 95% confidence intervals.

#### Pilot (retrospective) screening campaign

Through simulated ranking experiments, Truchon et al. demonstrated that the AUC ROC metric is dependent on the ratio of actives/inactives, and the standard deviation of the metric converges to a constant value when small yield of actives of the screened library (*Ya*, also called ratio of actives or prevalence of activity) are used (ratios of actives below 0.05 seem to provide more robust results). Reasonably small *Ya* also ensures that the saturation effect is constant or absent. A high number of decoys (around 1,000 or higher) contribute to a controlled statistical behavior, especially if poorly performing classifiers/methods are applied (Truchon and Bayly, 2007). Accordingly, we have performed pilot (retrospective) VS campaigns for the individual classifiers and classifier ensembles. For that purpose, we have dispersed the active compounds from each test set among a large number of decoys obtained with the help of the enhanced Directory of Useful Decoys (DUD-E) (Mysinger et al., 2012). Each of the test set active compounds from each dataset was used as a query in the DUD-E website, thus generating paired putative inactive compounds (decoys) for each of those active compounds. As a result, the pilot database contained 29 known active compounds dispersed among 1302 DUD-E decoys and 53 known inactive compounds, adding up a total of 1384 compounds and displaying a *Ya* of 0.021.

#### Building positivity predictive value surfaces and choosing an adequate score threshold value

A practical concern in virtual screening campaigns is to predict the actual probability that a predicted hit will prove truly active when submitted to experimental testing (the *PPV*). Estimation of such probability is however obstructed due to its dependency on the *Ya* of the screened library, which cannot be known *a priori*:

(1) $$PPV=\;\frac{\left(Se)(Ya\right)}{\left(Se\right)\left(Ya\right)+(1-Sp)(1-Ya)}$$

where *Se* represents the sensitivity associated to a given score cutoff value and *Sp* represents the specificity. Equation (1) was applied to build *PPV* surfaces. 3D plots illustrating the interplay between *PPV*, the *Se/Sp* ratio and *Ya* were built for each individual model and for each model ensemble. Using the corresponding pilot database (as described in previous subsections), *Se* and *Sp* were computed in all the range of possible cutoff score values. Note that there is no guarantee that the *Se* and *Sp* associated to each score value (and thus, the ratio *Se/Sp*) will be the same when applying the classifiers to other compound databases, e.g., in the real virtual screening campaign; nevertheless, since controlled statistical behavior is observed for database sizes of about 1000 compounds or more and *Ya* below 0.05, we can reasonably assume that the ROC curve and derived metrics will be similar when applying the models to classify other large chemical databases with low *Ya*. In order to build the *PPV* surfaces, and taking into consideration that in real VS applications *Ya* is ignored *a priori* but invariably low, *Ya* was varied between 0.001 and 0.010. The R package plotly (https://cran.r-project.org/package=plotly) was used to obtain all the *PPV* graphs. Visual analysis of the resulting *PPV* surfaces allowed us to select a score cutoff value with a desired range of *PPV*.

#### Virtual screening

Based on visual inspections of the resulting of *PPV* graphs, we have applied an 8-model ensemble using the MIN operator to combine individual models for virtual screening, choosing 0.354 as score threshold (above 0.354 compounds from the screened databases have labeled as predicted active compounds). Such threshold corresponds to a Se/Sp ratio of 0.666. It was checked that every hit belonged to the applicability domain of the model from the model ensemble that assigned the minimum score (which, was, according to our combination scheme, the one that ultimately decided if a compound was or was not labeled as an active). The leverage approach was used to assess if a hit belongs to the applicability domain, using 3*d*/*n* as cutoff value, where *d* is the number of descriptors in the correspondent model and *n* is the number of compounds in the training set.

We have used the 8-model ensemble to screen two databases: (a) DrugBank 4.0, an online database containing extensive information about the US Food and Drug Administration (FDA) approved, experimental, nutraceutical, illicit and investigational drugs (Law et al., 2014); (b) Sweetlead, a curated database of drugs approved by other international regulatory agencies, compounds isolated from traditional medicinal herbs, and regulated chemicals (Novick et al., 2013). These two databases were curated using Standardizer 16.10.10.0 (ChemAxon). We have applied the following actions to generate homogeneous representations of the molecular structure for the virtual screening: (1) Strip salts; (2) Remove Solvents; (3) Clear Stereo; (4) Remove Absolute Stereo; (5) Aromatize; (6) Neutralize; (7) Add Explicit Hydrogens; and (8) Clean 2D. Additionally, duplicate structures were removed using Instant JCHEM v. 17.2.6.0. Based in the results obtained and considering the most direct candidates for repositioning, two compounds were selected for experimental evaluation. Hits submitted to experimental testing were acquired from Sigma-Aldrich.

### Structure-based virtual screening

The 24 best solutions from ligand based virtual screening were submitted to molecular docking calculations. To this end we employed as target our 3D model of the putrescine permease *Tc*PAT12 previously constructed (Dietrich et al., 2018). This model was based on the arginine/agmatine antiporter (AdiC) from *E. coli* as template (Protein data bank accession code: 3L1L, Feng et al., 2000), which was identified through Blast sequence search and showed 23% of sequence identity and 64% coverage of the entire query sequence. The model of TcPAT12 architecture was achieved by Modeller Software (Webb and Sali, 2016). The best model has been selected based on the normalized Discrete Optimized Protein Energy parameter (z-DOPE) (Shen and Sali, 2006) and the GA341 score (which analyze the reliability of a model based on statistical potentials) (Melo and Sali, 2007). This structure had the highest number of residues in allowed area (96%) on Ramachandran plot, and none of the residues located in non-allowed regions were involved in the active site of the transporter.

The docking conditions were defined according to our previous studies, which pointed to Autodock4.2 (rigid mode) as the best software to discriminate known inhibitors from non-inhibitors of *Tc*PAT12 through the docking score (Dietrich et al., 2018). The “docking active site” was set according to previous research of Soysa et al. (2013). They proposed the location of the putrescine-binding pocket in a region that includes Gly69, Cys66, Trp241, Ala244, Asn145, Cys396, Asn245, Tyr148, Tyr400 amino acids. Autodock4.2 calculations were computed in a grid with the default spacing (0.375 A) between the 44 × 58 × 40 grid points in x, y, z directions respectively. Additionally, we performed the standard estimation for all the variables such as Marsilli-Gasteiger partial charges. We computed 100 docking runs for each compound, with a rigid target and flexible ligands (allowing the rotation of all non-ring torsion angles of the candidates).

### Experimental assays

#### Biological activity against different *T. cruzi* stages

For all assays, stock and working solutions of the candidate drugs were prepared using DMSO as solvent and test or control conditions were tested in triplicate.

Epimastigotes of the Y strain of *T. cruzi* were cultured at 28°C in BHT medium supplemented with 20 ug/ml haemin, 10% heat-inactivated fetal bovine serum (FBS), 100 μg/ml streptomycin and 100 U/ml penicillin. The antiproliferative activity of the candidates was tested at concentrations from 1 to 100 μM in cultures initiated at 10^7^ cells/ml. After 4 and 8 days, the number of viable parasites were counted using a hemocytometer chamber under the light microscope (Bellera et al., 2013). Controls were performed under the same culture conditions with equal concentrations of DMSO as for candidate drugs. The EC_50_ values were determined from dose response curves fitted to a sigmoidal equation (Boltzmann model) or extrapolated from linear fitting plots (Fernández et al., 2013).

*T. cruzi* trypomastigotes were purified at the parasitemia peak from peripheral blood of mice infected with the RA strain. Trypomastigotes (1 × 10^4^ per well) were cultured in a 96 well-plate (final volume 200 μl) in RPMI medium supplemented with 10% FBS at 37°C in 5% CO_2_ atmosphere. After 24 hours motile parasites were counted in hemocytometer chamber under the light microscope (Miranda et al., 2015). Results were expressed as % viability of trypomastigotes at 20 μM of the hits. Controls were performed under the same culture conditions with equal concentrations of DMSO as for candidate drugs. The negative control was cultured with PBS and the positive control was cultured with Benznidazole (20 μM).

*In vitro* evaluation of drug activity against intracellular amastigotes. J774 cells infected with bloodstream trypomastigotes of RA strain were cultured at 37°C in humidified incubator with 5% CO2 in 24-well plates (1.5 × 10^5^ cells per well; final volume of 500 μl in duplicate). After 24 h of incubation, increasing doses of freshly prepared dilutions of benznidazole, meclizine dihydrochloride, cinnarizine, or butoconazole were added at final concentrations of 5, 20, and 50 μM for meclizine, and cinnarizine and 1, 5, and 20 μM for butoconazole nitrate. Seventy-two hours later, medium was drained and cells were fixed for 10 min in ice-cold methanol and stained with 10% v/v of Giemsa solution for 15 min. The number of amastigotes per 100 host cells was recorded. Control cultures were incubated in medium alone or with equal DMSO concentrations. Citotoxicity was analyzed in trypsinized cell suspensions, after addition of Propidium iodide (PI) (Sigma, St. Louis, USA) (5 μg/ml) 10 min prior to analysis by fluorescence and light microscopy of the number of viable and dead cells.

#### Aminoacid/polyamine transport assay

Aliquots of *T. cruzi* epimastigotes (3 × 10^7^ parasites) starved for 3 hs in 2% glucose—phosphate-buffered saline (PBS) were collected, centrifuged at 1,500 *g* for 10 min and washed three times with PBS. Cells were then resuspended in 2 ml of PBS containing 5 μM (^14^C)-putrescine, or (^14^C) arginine, and candidates at a final concentration of 50 μM previously solubilized in DMSO. Aliquots were taken at different time points, centrifuged and washed three times with 1 ml of ice cold 10 mM solution of unlabeled putrescine/arginine in PBS. Pellets were resuspended and radioactivity determined in UltimaGold XR (Díaz et al., 2014). All experiments were performed in triplicate.

The effect of the candidate on parasite viability under uptake assay conditions was evaluated through the tetrazolium salt (MTT) reduction assay (Mosmann, 1983). Briefly, 10 μl of 5 mg/ml MTT dye (3[4,5-dimethylthiazol-2-yl]-2,5-diphenyltetrazolium bromide) was added to the eppendorf tubes containing 10^6^ parasites in 100 μl of BHT and the drug candidates at 50 μM solubilized in DMSO. After incubation for 3 h at 28°C, the tubes were spin-dried (3,000 rpm) and the pellet with the formazan crystals were dissolved with 100 μl of DMSO. The optical density (OD) was determined using a microplate reader (Labsystems Multiskan MS, Finland) at 570/695 nm. Under such conditions, the OD is proportional to the viable cell number in each well. All experiments were performed in triplicate.

## Results

### Model development and validation and virtual screening

Two serial virtual screening methods were applied to find out putative *Tc*PAT12 inhibitors. First, we resorted to a computationally inexpensive ligand-based approach. For that purpose, 1,000 individual linear classifiers were obtained by applying a random subspace approximation. The individual models were externally validated by using an independent test set and, for a more challenging and realistic simulation, by retrospective screening of a simulated library where a small proportion of active compounds was dispersed among a high number of (mostly putative) decoys. 82, 57, and 25% of the individual classifiers displayed AUC ROCs above 0.8 for the training set, the test set and the simulated DUD-E database, in that order. This suggests some degree of overfitting and corroborates that the pilot (retrospective) screening campaign against the DUD-E database is the more challenging task for the classifiers. Table 1 shows the eight individual classifiers that showed the best performance on the training and test sets, and also on the DUD-E database.

**Table 1:** Values of the AUROC metric for the best eight individual models and the best 8-model ensemble.

**Model**	**Training set**	**Test set**	**DUD-E database**
8-MODEL ENSEMBLE (MIN)	0.851 (±0.0281)	0.885 (±0.0367)	0.976*** (±0.0085)
8-MODEL ENSEMBLE (RANKING)	0.886 (±0.0239)	0.878 (±0.0375)	0.976*** (±0.0082)
8-MODEL ENSEMBLE (AVERAGE)	0.891 (±0.0234)	0.887 (±0.0357)	0.970*** (±0.0096)
8-MODEL ENSEMBLE (VOTING)	0.833 (±0.0283)	0.810 (±0.0516)	0.959* (±0.0173)
348	**0.882 (**±**0.0250)**	**0.885 (**±**0.0360)**	**0.934 (**±**0.0123)**
706	0.809* (±0.0319)	0.736** (±0.0565)	0.924 (±0.0205)
981	0.843 (±0.0298)	0.837 (±0.0467)	0.922 (±0.0254)
557	0.778** (±0.0343)	0.818 (±0.0482)	0.919 (±0.0203)
123	0.850 (±0.0285)	0.882 (±0.0382)	0.918 (±0.0185)
693	0.860 (±0.0280)	0.828* (±0.0459)	0.913* (±0.0171)
560	0.775** (±0.0348)	0.779* (±0.0525)	0.911* (±0.0185)
746	0.844 (±0.0288)	0.820 (±0.0456)	0.910 (±0.0195)

AUROCs statistically different from the correspondent column for the best individual model (model 348).

* p < 0.05,

** p < 0.01, and

*** p < 0.001.

*The highest AUC for an individual model is indicated in bold*.

The best individual model included the following features:

Model 348: Class = 3.12777 + 0.03474 ^*^ F06[C-C] + 0.20805 ^*^ S-107 – 0.04291 ^*^ F05[N-N] + 0.39611 ^*^ C-039 – 0.34582 ^*^ SM5_B(s) + 6.25705 ^*^ Eta_epsi_A + 0.53013 ^*^ nSO2OH – 1.28338 ^*^ SpMax_H2 + 0.44827 ^*^Eig04_AEA(ri) – 1.73390 ^*^ ATSC1e + 0.05975 ^*^ CATS2D_09_PL + 0.02805 ^*^ SaaO
Wilks' Lambda: 0.59
*F*_(12, 161)_ = 9.23
*p* < 0.0000

Dragon's nomenclature for the molecular descriptors has been kept in the previous expression. F06[C-C] refers to the count of C-C at a topological distance of 6; S-107 represents the count of R2S/RS-SR groups; F05[N-N] stands for the frequency of N - N at a topological distance of 5; C-039 refers to Ar-C(= X)-R groups; SM5_B(s) corresponds to the spectral moment of order 5 from the Burden matrix weighted by I-State; Eta_epsi_A is the eta average electronegativity measure; nSO2OH stands for the number of sulfonic (thio-/dithio-) acids; SpMax_H2 is the leading eigenvalue from reciprocal squared distance matrix; Eig04_AEA(ri) denotes the fourth eigenvalue from augmented edge adjacency matrix weighted by resonance integral: ATSC1e corresponds to the Centred Broto-Moreau autocorrelation of lag 1 weighted by Sanderson electronegativity; CATS2D_09_PL is the CATS2D Positive-Positive at lag 09 and; SaaO corresponds to the sum of aaO E-states.

**Figure 3:**
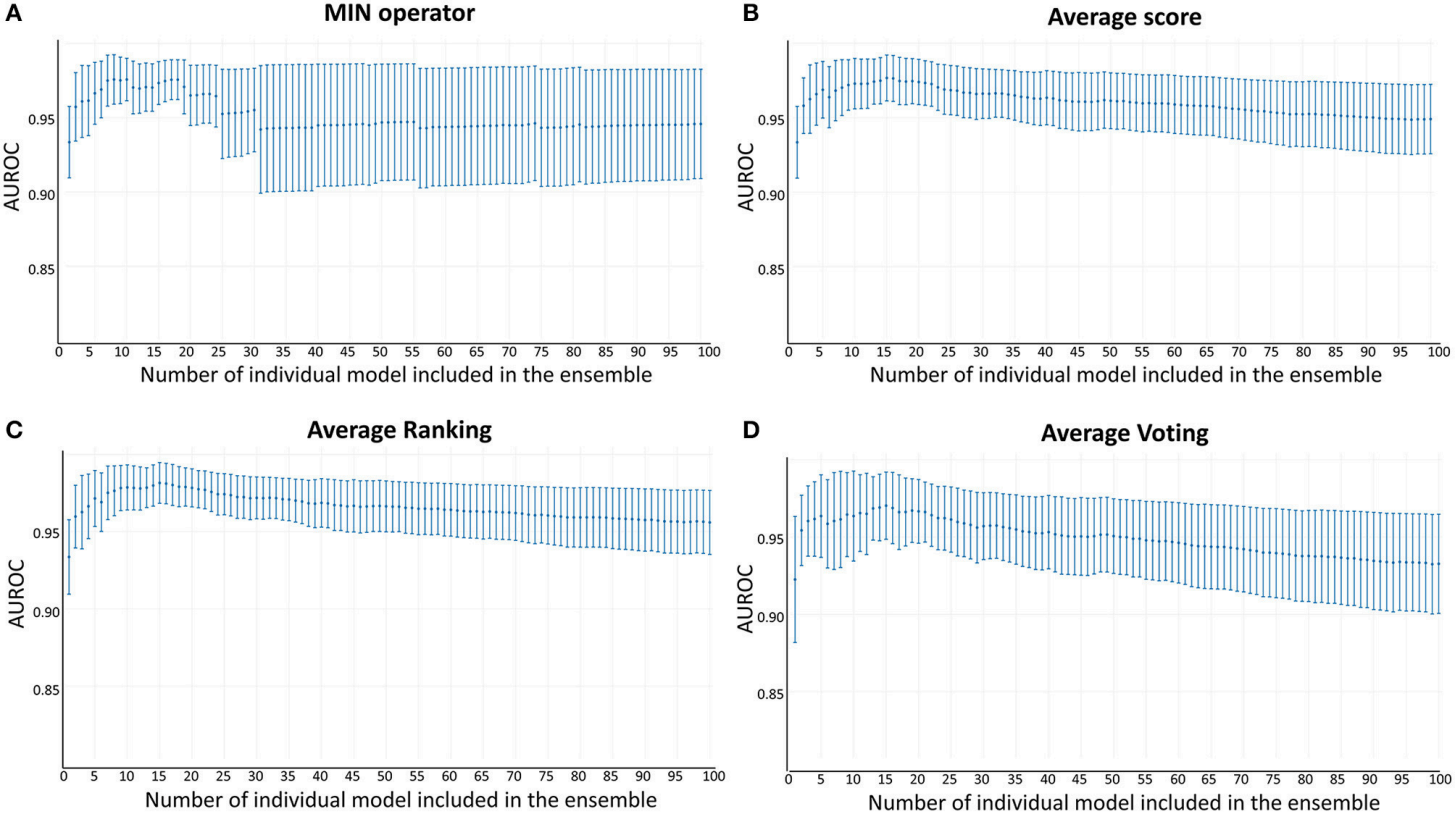
AUROC metric vs. the number of combined models in the DUD-E database **(A)** MIN operator; **(B)** Average score; **(C)** Average Ranking; **(D)** Average Voting.

Whereas the performance of the best individual classifiers was quite satisfactory, we resorted to ensemble learning to obtain meta-classifiers with improved accuracy and a more robust behavior. The expectations on the model combination approach were confirmed statistically: no matter which combination scheme is applied, the model ensembles show a statistically similar behavior to the best individual model when classifying the training and test set compounds, while statistically outperforming the individual models when considering the DUD-E database (*p* < 0.05 in all cases). The MIN, RANKING and AVERAGE combination schemes led to the best results (Table 1), with *p*-values below 0.001. When considering the influence of the number of combined models on the AUROC metric, it was observed that although the ensemble learning approach always seems to improve the results in comparison with the individual classifiers, combinations above 10 models tend to poorer behavior in terms of AUROC values but also regarding the standard deviation of the mean, which increases with the number of models included in the ensemble (Figure 3).

**Figure 4:**
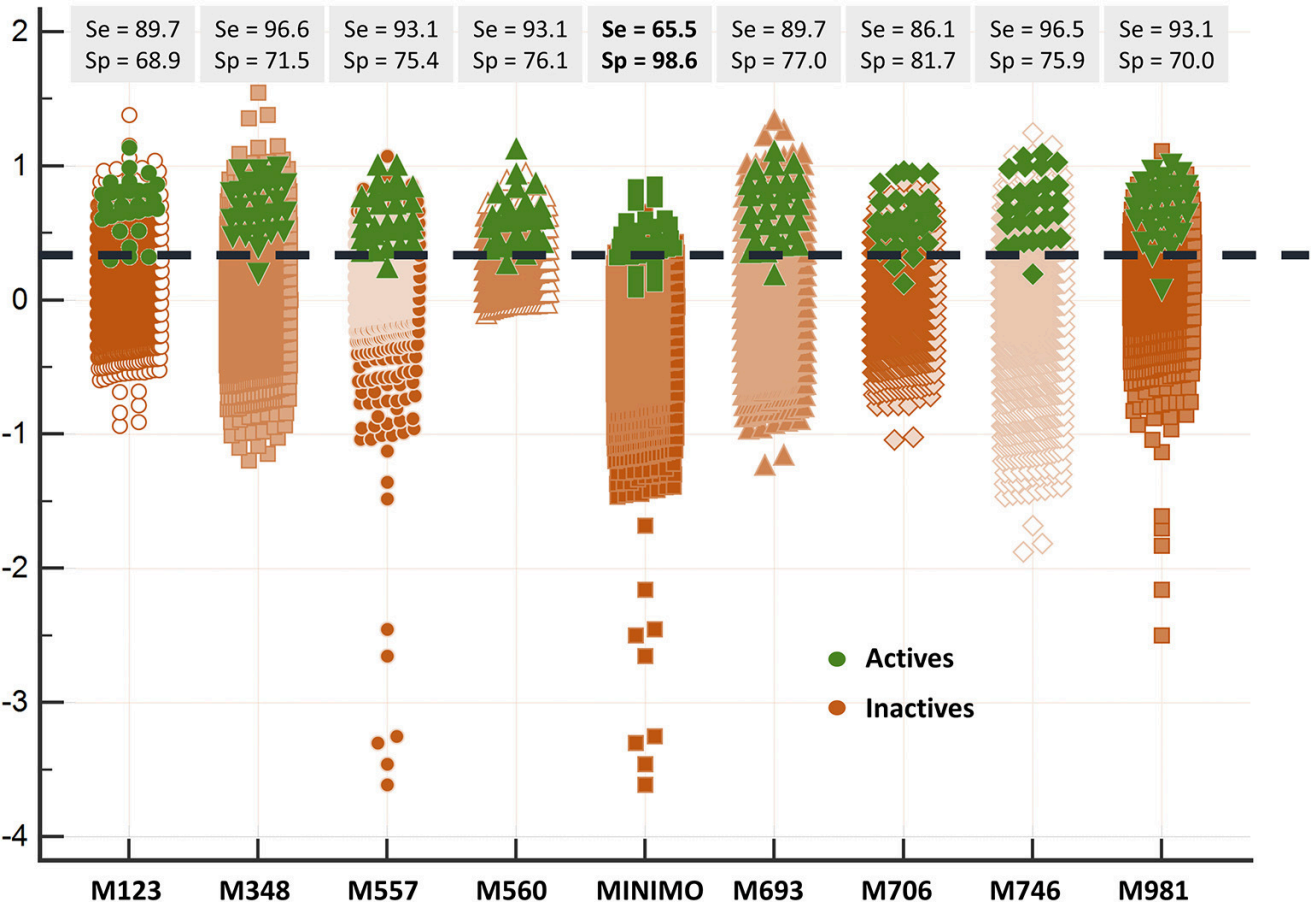
Discriminating abilities of the individual models and the best model ensemble against the DUD-E database. The MIN combination scheme clearly improves Sp (note the enhanced separation of the inactives from the actives in comparison with individual models).

Since all the model combination approaches exhibited similar behavior against the test set and the DUD-E database for relatively low number (below 10) of combined classifiers, we chose to move to the real virtual screening campaign with the combination scheme based on the MIN. In our experience, this is the most conservative approach which tends to display the smallest false positive rate. This was confirmed when comparing Sp and Se of the 8-model meta-classifier with those of the best performing individual models, where a substantial improvement in Sp was observed (Figure 4). This is a particularly good result in our context (small academic group from a mid-income country, with limited resources to invest in hit experimental validation); we often prefer to reduce the false positive rate even if this means losing sensitivity and sacrificing some active scaffolds. However, in this particular case we have chosen to refine the former criteria (prioritizing Sp) by resorting to what we have called PPV surfaces. With the help of these surfaces, the evolution of the most relevant metric for our purposes, the PPV value, can be visually (or, eventually, mathematically) optimized as a function of the Se/Sp ratio across a range of *Ya* values. For this analysis, we have used the association between Se/Sp and score values that have been observed in the pilot screening campaign. The strongest assumption of our approach is that the Se/Sp value observed for a given score during the pilot screening campaign against the DUD-E database will hold when screening other databases (e.g., the ones screened in real virtual screening applications). This is of course not necessarily true. However, since the AUROC values obtained for the DUD-E database are unequivocally high (always above 0.9 for the individual models and very close to the perfect value of 1 for the ensembles) while on the other hand the DUD-E database *Ya* ratio (quite below 0.05) and size (>1,000 compounds) speak of a controlled statistical behavior, we believe it is a reasonable assumption in the present setting.

**Figure 5:**
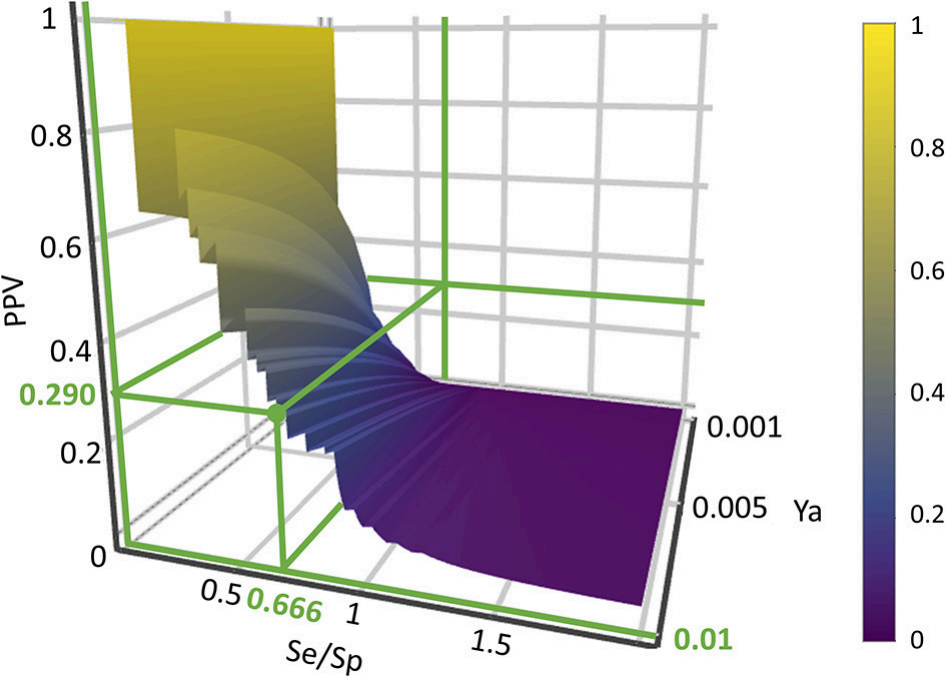
PPV surface for the best 8-model ensemble.

When analyzing the PPV surface for the 8-model combination based on the MIN operator, it was observed that in the current scenario favoring Sp over Se has a positive impact on the PPV, thus resulting in higher probabilities of confirming *in silico* hits submitted to experimental validation (Figure 5). It should be emphasized that such behavior is not general: other situations linked to different PPV surface shapes might lead to an entirely different conclusion (that is, the need to prioritize Se over Sp to enhance PPV). The selection of the best cutoff value should be based in the specific PPV surface obtained in each particular modeling situation.

Using the PPV surface, we chose 0.354 as score threshold to be used in our real virtual screening campaign; such score is associated to a Se/Sp ratio of 0.666 for the 8-model ensemble based on the MIN operator. Whereas higher scores linked to lower Se/Sp ratio would, according to the PPV surface, result in improved PPVs, the resulting number of hits would also be substantially diminished, leaving very few choices for subsequent acquisition and experimental testing (the ligand-based screen using the 0.354 cutoff score resulted in only 24 hits, with just 15 of them being approved drugs, the most straightforward candidates to drug repurposing).

The 24 hits emerging from the ligand-based screening stage were submitted to the structure-based screening (docking into the *Tc*PAT12 homology model), with 17 of them surviving the docking stage and only 9 of them having achieved approved status. Table 2 shows the hits selected through the combined ligand- and target-based approach, including the PPV range for the correspondent score value of the 8-model ensemble between Ya values of 0.001 and 0.010. Note that 3 of the hits, namely clomifene, oxiconazole and clofazimine have previously been assayed against *T. cruzi*, with positive results (Sykes and Avery, 2013; Bellera et al., 2015; Kaiser et al., 2015). Most of the compounds have a docking score lower than the value found for the natural ligand putrescine (−**6.0**), which was calculated previously in the same docking conditions. The results justify the selection of the structures as promising candidates. The exception was clofazimine, with a docking score of −1.58. In this particular case, the drug has been identified as trypanocidal agent within our research group, during an *in silico* screening to detect novel cruzipain inhibitors. The drug later confirmed its potential both in acute and chronic rodent models of Chagas disease (Bellera et al., 2015; Sbaraglini et al., 2016). In that occasion, though, it was observed that the potency of the drug against the parasite was higher than the inhibitory potency against cruzipain, suggesting multiple mechanisms of action besides cruzipain inhibition.

**Table 2:** Candidates selected through the combined ligand- and target-based approach.

**Name**	**MIN Score**	**PPV% (Ya = 0.001)**	**PPV% (Ya = 0.01)**	**Structure**	**Score docking**	**Current therapeutic indication**
Clomifene	0.4837	14.64	63.38	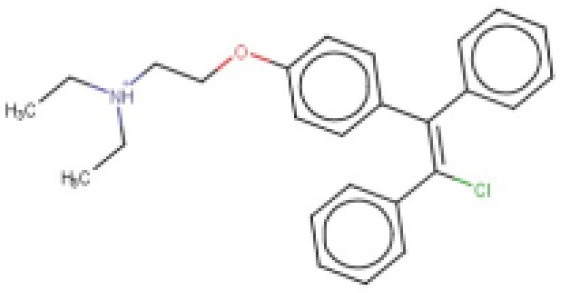	−9.37	Used mainly in female infertility due to anovulation
Butoconazole	0.4768	14.64	63.38	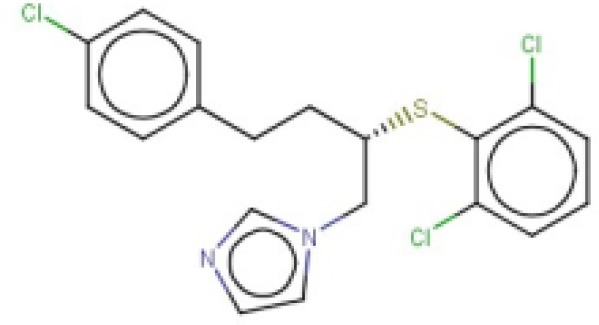	−9.87	Local treatment of vulvovaginal candidiasis
Meclizine	0.4546	12.30	58.61	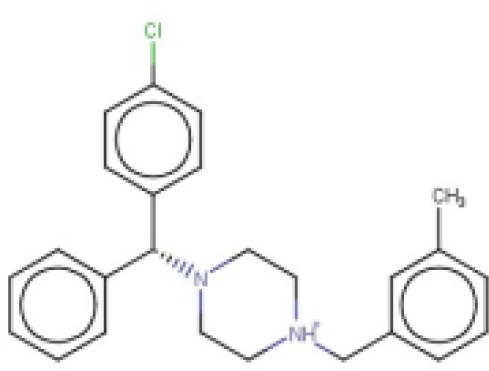	−8.95, −6.64^a^	Motion sickness and vertigo
Clemizole hydrochloride	0.4544	12.30	58.61	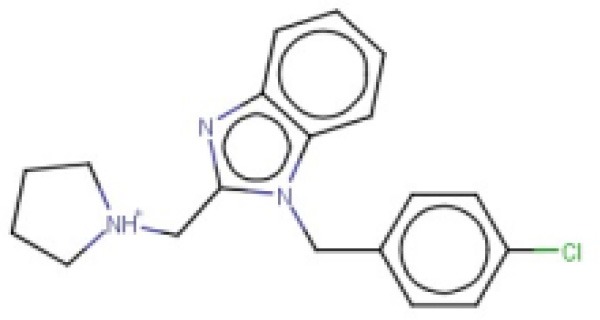	−8.91	Allergies
Cinnarizine	0.4273	9.20	50.56	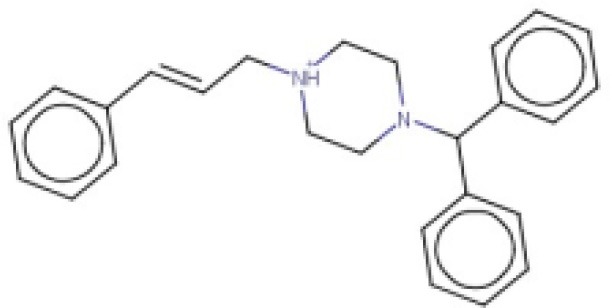	−7.43	Motion sickness and vertigo
Centchroman	0.3798	5.67	37.76	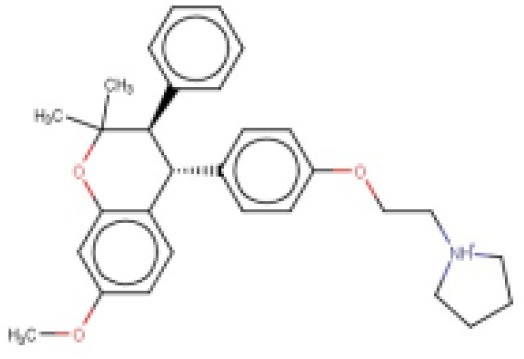	−6.31	Primarily used as a contraceptive
Oxiconazole	0.3751	5.00	34.68	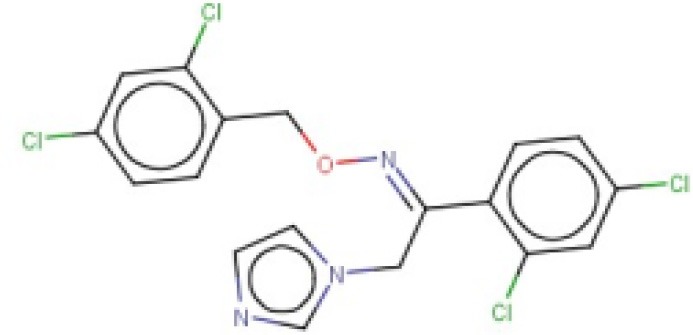	−10.44	Dermal fungal infections.
Astemizole	0.3666	4.25	30.96	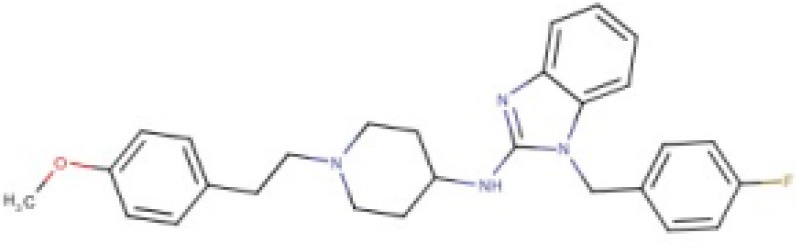	−6.45	Allergies
Clofazimine	0.3605	4.06	29.92	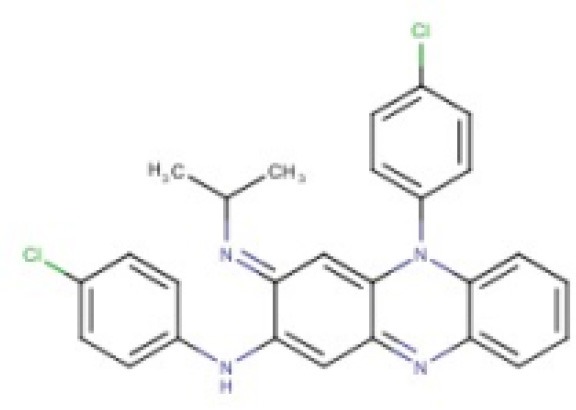	−1.58	Leprosy

a *The docking scores of both isomers were calculated*.

**Figure 6:**
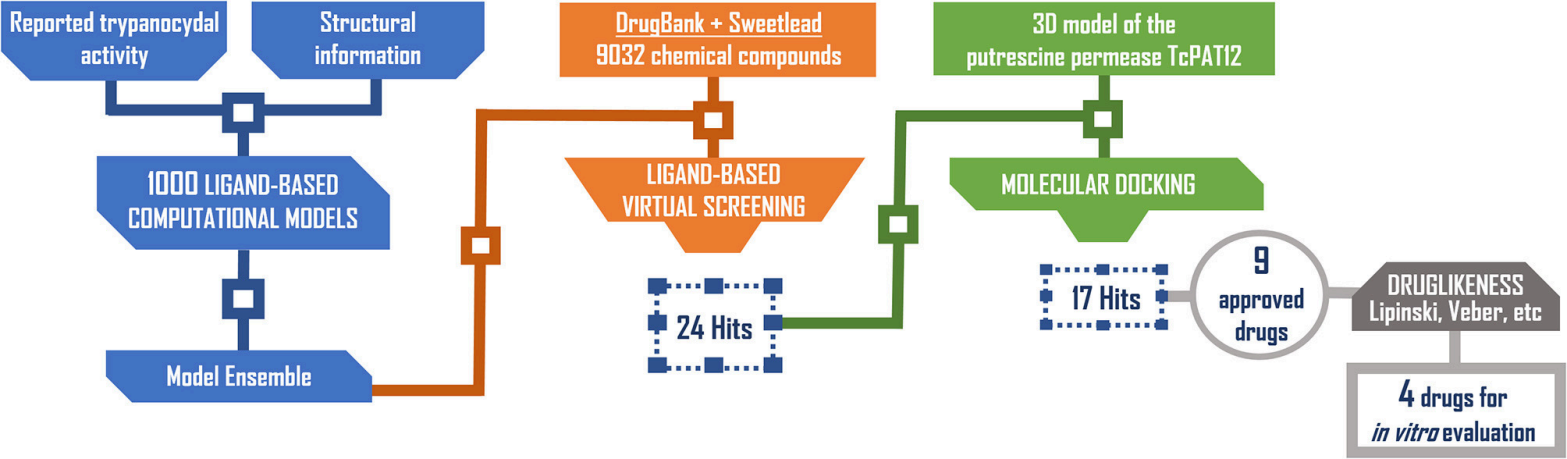
Screening workflow used in the present study. The number of compounds retained at each step for further studies is shown.

Based on access to the chosen compounds, we decided to test clofazimine, cinnarizine, meclizine dihydrochloride, and butoconazole nitrate (Sigma Aldrich) effects on putrescine uptake in *T. cruzi*. Cinnarizine, meclizine, and butoconazole were also tested against *T. cruzi* epimastigotes, trypomastigotes, and amastigotes. The latter assays were omitted for clofazimine, since as already discussed, this drug had previously been tested against the different *T. cruzi* stages, with positive results. Before acquisition, it was verified if the compounds obeyed different druglikeness rules. All compounds satisfy Lipinski's rules (they satisfy three out of four of the Lipinski's proposition, only violating the proposition linked to clogP), Veber's rule and the druglikeness criteria adopted by Monge et al. in previous studies (Lipinski et al., 2001; Veber et al., 2002; Monge et al., 2006). All in all, these analysis suggest that they have high probabilities of displaying good oral bioavailability (Lipinski's and Veber's rules) while also excluding some undesirable properties such as highly reactive chemical groups and possible toxicophores) (Monge's criteria). This is not a surprising result since all the hits submitted to experimental validation are approved drugs. A summary of the screening workflow and the number of hits surviving each step is presented in Figure 6.

### Experimental testing

**Figure 7:**
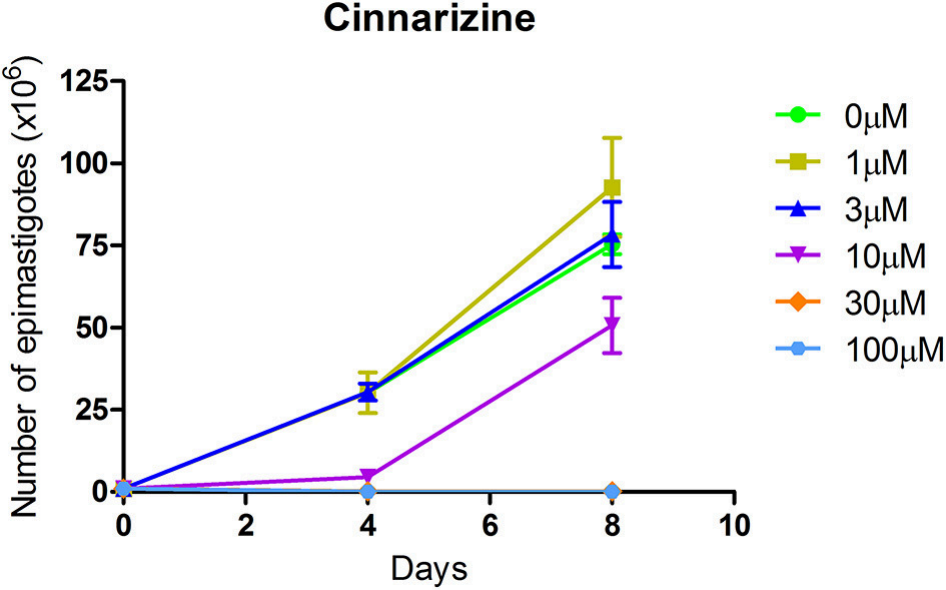
Effect of cinnarizine on *T. cruzi* epimastigotes proliferation. Results are expressed as the mean ± SD of triplicate experiments.

**Figure 8:**
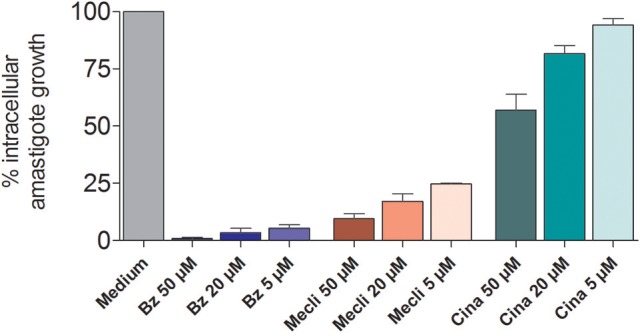
Inhibitory effects of the three assayed hits against *T. cruzi* amastigotes.

The effect of different concentrations of cinnarizine, meclizine and butoconazole against *T. cruzi* epimastigotes was tested (Figure 7) and the correspondent EC_50_ was calculated. The three drugs showed a clear inhibition in a dose-depend manner on the proliferation of epimastigotes, with a EC_50_ (at day 4) of 6.05 μM, 8.39 μM and 3.08 μM, respectively. The reference drug benznidazole displayed a EC_50_ of 2.56 μM against epimastigotes. When tested against trypomastigotes at 20 μM, cinnarizine displayed a slight inhibition in viability (30%). No inhibition was observed for the other two hits tested. When testing its effects against amastigotes, butoconazole showed a considerable cytotoxicity against J774 cells at all the assayed concentrations (100.0%; 57.5 ± 0.6%; 48.3 ± 6.3% at 50 μM; 20 and 5 μM respectively), and it was therefore disregarded for future investigations. Meclizine showed cytotoxicity against J774 cells at 50 μM (13.9 ± 2.0%), though no toxic effects were observed at lower concentrations. Cinnarizine showed no toxicity at any of the tested concentrations. Meclizine significantly reduced the number of amastigotes per 100 cells at the three assayed concentrations. Cinnarizine showed a weak effect against amastigotes, displaying almost a 50% inhibition at 50 μM (Figure 8).

**Figure 9:**
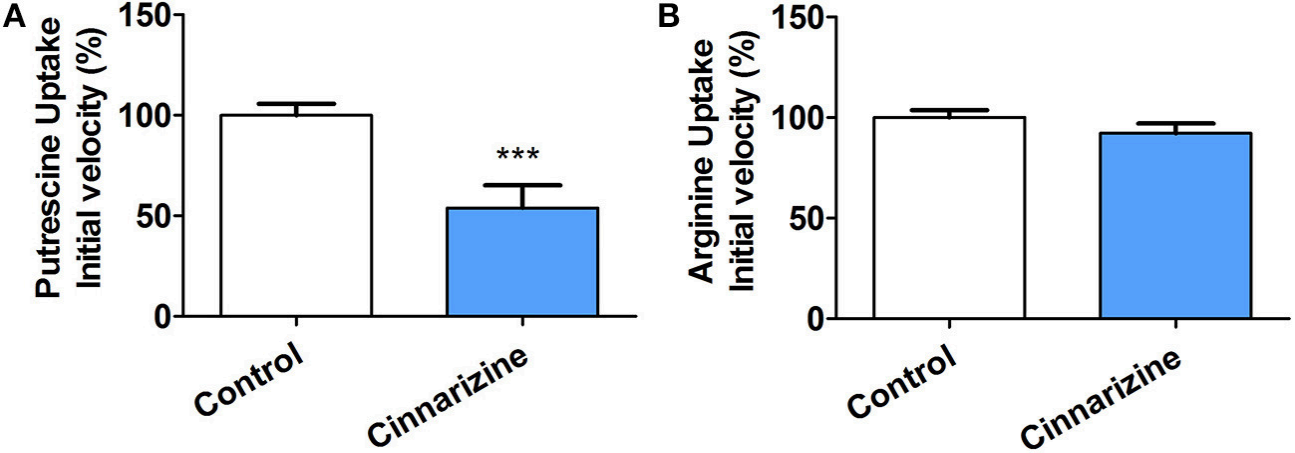
Effect of 50 μM cinnarizine and clofazimine on putrescine **(A)** and arginine **(B)** uptake in *T. cruzi* epimastigotes. Values are expressed as % mean ± SD in comparison with control. Statistical analysis was performed by one-way ANOVA test followed by a *post-hoc* Dunnett's multiple comparison test (***p < 0.005).

To determine if the mechanism of action of the candidates correlates with that predicted by our models, the inhibition of putrescine uptake by *T. cruzi* epimastigotes was determined (Figure 9A). A 10-fold molar excess of the candidate drugs were tested. Cinnarizine and clofazimine showed a clear effect on putrescine uptake with a significant initial velocity reduction to 52.56 ± 4.84 and 30.85 ± 2.74% respectively, compared with transport in control conditions. In contrast, meclizine and butoconazole did not display any inhibitory effect on putrescine uptake, which suggests that their trypanocidal effect is related to other mechanisms of action. Meclizine has previously been shown to inhibit cruzipain at low μM concentrations (Doak et al., 2010).

The specificity of the putrescine uptake inhibition by cinnarizine and clofazimine was checked by testing the effect of both active hits on other transporter of the same family (Figure 9B). The MTT assay indicated that under the uptake conditions (with 50 μM of the drug and 1% DMSO), neither cinnarizine nor clofazimine presented a cytotoxic effect on the parasites (not shown).

## Discussion

### Comparison with previous studies

Whereas studies to exploit polyamine transporters as molecular drug targets for the development of new trypanocidal agents are at an early stage, some interesting considerations may be outlined from the considering the present study and the few previous reports describing the screening of new inhibitors of *T. cruzi* putrescine uptake. First, some of the hits identified in earlier studies have shown to be more effective against *T. cruzi* trypomastigotes than against other stages of the parasite (Reigada et al., 2017, 2018). For instance, the reported IC_50_ of isotretinoin against trypomastigotes (130 nM) is about 230-fold lower than the one against epimastigotes (Reigada et al., 2017). The opposite was observed in this study for cinnarizine, since epimastigotes were more sensitive to the drug than trypomastigotes. There are some possible explanations to these varying degrees of sensitivity to putrescine uptake inhibition across *T. cruzi* stages. It is possible that different forms of the parasite resort to different primary transport mechanisms of polyamines (each of them with different drug specificities) (Seguel et al., 2016). A similar possibility has been suggested in *Leishmania*, where it has been observed that amastigotes and promastigotes use different transporters for polyamine uptake (Müller et al., 2001; Colotti and Ilari, 2011).

The comparison of the present study with previous *in silico*-guided drug repurposing campaigns targeting *T. cruzi* polyamine uptake is hindered by the fact that very few earlier studies exist and that different virtual screening approaches have been applied in them. The first report of a virtual screening application to identify putrescine uptake inhibitors came from Alberca et al. who back in 2016 used an ensemble of six linear classifiers and identified three novel confirmed hits (out of five hits submitted to experimental screening): sertaconazole, triclabendazole, and paroxetine (Alberca et al., 2016). The same 6-model ensemble was later applied in parallel with a similarity-based screen and in sequence with target-based screening, with one out of four hits validating their predicted activity *in vitro* (Dietrich et al., 2018). For their part, Reigada et al. used a combination of similarity-based virtual screening and molecular docking, obtaining two experimentally confirmed hits (isotretinoin and acitretin) out of a total of three tested compounds (Reigada et al., 2017). The statistical comparison of the true PPV (confirmed hits over total number of hits tested) in the aforementioned virtual screening campaigns has no point due to the small number of hits tested in each occasion. Nevertheless, such PPV was in all cases, including the current study, well above the median value of hit rate in virtual screening studies (13%), as reported in a critical literature analysis of virtual screening results published between 2007 and 2011 (Zhu et al., 2013).

**Figure 10:**
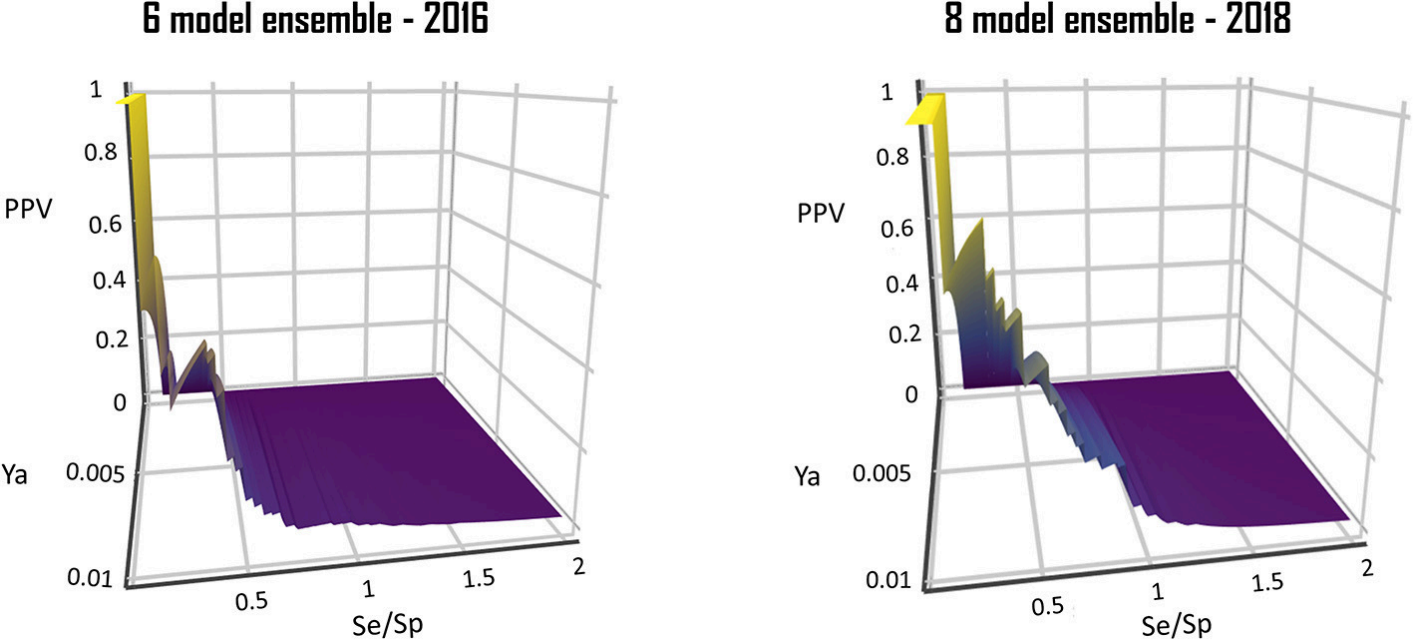
Comparison of the PPV surface of the 8-model ensemble of classifiers reported here with that of the 6-model ensemble reported back in 2016.

From a visual comparison of the (theoretic) PPV surfaces of the 6-model ensemble reported by Alberca et al. (2016) and the currently reported 8-model ensemble, it is clear that the 8-model ensemble reported here shows a more robust and predictable behavior in the *Se/Sp* range that goes from 0.3 to 1.0. (Figure 10).

### The power of ensemble learning

The DUD-E database used as a validation tool in our retrospective virtual screening campaign confirmed that retrospective screening is the most challenging task (besides prospective screening) for a model conceived for *in silico* screening applications, as in the case of individual classifiers, the AUROC tends to fall sharply when progressing from the training set to the DUD-E database. In fact, whereas an astonishing 82% of the individual models achieved remarkable AUROCs (above 0.8) when confronted to the training set, the percentage of individual models that obtains such achievement against the DUD-E falls to only 25%.

Echoing previous studies, though, the ensemble learning approximation led to more robust results, improving accuracy in the predictions and generalization (which, in our case, is reflected by an improved behavior on the test set and the DUD-E database).

### Do we have good repurposed candidates?

Despite two out of four hits did not confirm inhibitory effects on putrescine uptake, all of them did confirm their trypanocidal effects using concentrations in the low μM range. However, does this mean that they are good candidates for drug repurposing?

Not necessarily. Cinnarizine, meclizine, and butoconazole all belong to therapeutic classes that have shown potential as antichagasic therapies in the past: calcium channel blockers (Engel et al., 2010; Benaim and Paniz Mondolfi, 2012; Planer et al., 2014), antihistaminic drugs (Engel et al., 2010; Planer et al., 2014; Lara-Ramirez et al., 2017) and antifungals (Lepesheva et al., 2011).

Butoconazole, however, is not a straightforward candidate for repositioning, since it is only used topically as antifungal. Accordingly, most of the advantages of drug repurposing will be lost if the second indication (in this case, Chagas' disease chemotherapy) requires systemic administration (Oprea and Overington, 2015). Furthermore, our experimental evidence of cytotoxic activity discourages further investigation. The advantages of drug repositioning will also be mostly lost if the dose required for the second indication is above the dose range clinically used for the original one (Oprea and Overington, 2015), as would probably be the case for cinnarizine and meclizine, which are currently administered in low daily doses. This is especially true in the case of cinnarizine, which showed only weak activity against the clinically relevant forms of *T. cruzi*. The scenario is further complicated by the fact that free drug exposure will be diminished by plasma protein binding: in the case of cinnarizine, for instance, around 90% of the drug in plasma is bound to plasma proteins.

The previous analysis does not imply that is impossible to efficaciously repurpose meclizine (which showed a very interesting effect against amastigotes, inhibiting 75% growth at only 5 μM concentration) or that both cinnarizine and meclizine are not useful as starting points for hit-to-lead programs but, in any case, many of the advantages of the strategy (i.e., bypassing some preclinical and clinical studies) will probably be lost.

## Conclusions

A cascade virtual screening approach comprising an ensemble of ligand-based classifiers and structure-based screening has been applied, identifying two new inhibitors of putrescine uptake in *T. cruzi* and reporting, for the first time, the trypanocidal effects of butoconazole (and antifungal) and cinnarizine and meclizine, two antihistaminic agents of the diphenylmethylpiperazine group commonly used to treat motion sickness and balance disorders. Interestingly, neither cinnarizine nor clofazimine modified arginine uptake by another member of the putative amino acid transporter (*Tc*PAT) family.

This is, to our knowledge, the first report implementing PPV surface analysis to select the score value to be applied in a virtual screening campaign.

## Ethics statement

All animal procedures were approved by institutional regulations of the Committee for the Care and Use of Laboratory Animals of the Universidad de Buenos Aires (Buenos Aires, Argentina) in accordance with government regulations [SENASA, resolution No. RS617/2002, Argentina]. All procedures were performed in accordance with institutional safety procedures.

## Author contributions

LA and JM have built and validated (*in silico*) ligand-based models and model ensembles to identify putrescine uptake inhibitors, under the supervision of AT. They later performed the first *in silico* screening step and data analysis related to ligand-based VS. RD and PP have applied structure-based filters (molecular docking) in the virtual screening campaign. MS, MR, LF, AP, and CM have performed the *in vitro* assays against the different *Trypanosoma cruzi* stages. LA and MR have performed the transport assays. They work in CA and CC labs, under their supervision. LA, MS, CC, CA, PP, and AT have also been actively engaged in manuscript preparation and discussion. All authors took part in the result discussion for the revised version.

### Conflict of interest statement

The authors declare that the research was conducted in the absence of any commercial or financial relationships that could be construed as a potential conflict of interest.

## Supplementary material

Data Sheet S1 Inactive dataset compounds.

Data Sheet S2 Active dataset compounds.
